# From guidelines to bedside - insomnia treatment practices in South Korea: a nationwide cohort study

**DOI:** 10.3389/fpsyt.2024.1453550

**Published:** 2024-09-13

**Authors:** Daa Un Moon, Zhaoyan Piao, Do Hyun Lee, Euna Han

**Affiliations:** ^1^ Department of Psychiatry and Psychotherapy, Charité Campus Mitte, Charité – Universitätsmedizin Berlin, Corporate Member of Freie Universität Berlin, Humboldt-Universität zu Berlin, and Berlin Institute of Health, Berlin, Germany; ^2^ College of Pharmacy, Yonsei Institute of Pharmaceutical Sciences, Yonsei University, Incheon, Republic of Korea

**Keywords:** insomnia, sleep disorder, cognitive behavioral therapy for insomnia, CBT-I, NHIS-NSC, nationwide cohort study

## Abstract

**Background:**

Insomnia is a prevalent disorder that impacts quality of life and leads to significant economic costs. Treatment includes both non-medication and pharmacological interventions, with international guidelines recommending cognitive behavioral therapy for insomnia (CBT-I) as the first-line treatment.

**Objective:**

To describe current insomnia treatment practices in South Korea, focusing on pharmacological and non-medication treatments, and to identify gaps in guideline implementation.

**Methods:**

This cohort study used data from the Korea National Health Insurance Service-National Sample Cohort (NHIS-NSC) from 2002 to 2019 and identified 18,003 patients newly diagnosed with insomnia between 2015 and 2019. This study analyzed treatment patterns and utilization rates.

**Results:**

Of the 18,003 patients, 16,181 (89.9%) received pharmacological treatment, resulting in 35,638 prescriptions. Zolpidem (60%) and benzodiazepines (30-40%) were the most prescribed medications. Most patients were treated in clinics, with consistent dosages and increasing treatment lengths. Psychotherapy claims rose from 3.20% in 2015 to 9.14% in 2019, particularly in general hospitals (22.06% to 48.37%), but remained low in clinics (1.26% to 2.08%).

**Conclusion:**

Pharmacological treatments dominate insomnia management in South Korea, with CBT-I being underutilized. Future efforts should focus on integrating non-pharmacological treatment into routine practice and exploring treatment risks and effectiveness based on patient demographics.

## 1 Introduction

Insomnia is clinically diagnosed based on complaints of difficulty falling asleep, staying asleep, or waking up too early, coupled with resulting daytime dysfunction, despite having adequate opportunity for sleep (1, 2). This condition is prevalent, affecting approximately 10% of the general population (3, 4), with current estimates ranging up to 20% (5). In South Korea, the prevalence of sleep disorders has significantly increased, rising from 8% in 2011 to 14% in 2020, nearly doubling over a decade (6). Insomnia is a recognized risk factor for several serious health issues, including depression, anxiety disorders, substance use disorders, suicidality, hypertension, and metabolic syndrome, and it significantly impacts quality of life (7–14). The functional consequences of insomnia are considerable, leading to reduced productivity, increased absenteeism, increased use of healthcare services, and a greater risk of accidents, all of which contribute to substantial economic costs (15, 16).

When a patient is diagnosed with insomnia, treatment can be initiated with a variety of interventions categorized broadly into non-medication treatments and pharmacological therapies. General management includes addressing comorbid medical and psychiatric conditions, modifying sleep-interfering medications and substances, and optimizing the sleep environment (17, 18). International guidelines recommend cognitive behavioral therapy for insomnia (CBT-I) and insomnia medications as effective specific treatments for insomnia disorder (19–23).

Non-pharmacological treatment options for insomnia include relaxation therapy, biofeedback, and cognitive behavioral therapy for insomnia (CBT-I). Among the non-pharmacological treatment options, CBT-I is the most empirically supported and widely used. It is recommended by several clinical guidelines from South Korea, the USA, and Europe as the first-line treatment for adults with chronic insomnia (19–23). Typically delivered over four to seven sessions, it includes sleep hygiene education, cognitive restructuring, and behavioral interventions such as bedtime restriction and stimulus control (17, 24). Meta-analyses have demonstrated that CBT-I leads to significant improvements in insomnia symptoms, sleep efficiency, and sleep quality across a wide array of clinical populations (25–28). CBT-I has been shown to be equally effective in the short-term as medication-based therapies, with greater long-term maintenance of positive effects and minimal side effects (17, 27). It has been provided through various methods, such as individual and group in-person sessions, as well as digitally delivered therapy. Overall, all these approaches are effective, although some research indicates that face-to-face therapy has better outcomes (29–31). In July 2018, the South Korean Ministry of Health and Welfare implemented a policy change, expanding the national health insurance coverage to include various forms of psychotherapy, such as CBT. However, the lack of formal training, awareness among healthcare professionals, and availability of clinical staff limits the use of non-medication strategies such as CBT-I (32–35).

Pharmacological treatments for insomnia are widely accessible and often prescribed due to their immediate effect on sleep symptoms and ease of prescription (18). Commonly used medications include benzodiazepines and Z substances (36). However, these medications are associated with substantial adverse events, such as sedation, psychomotor impairment, and potential for abuse (18). Additionally, hypnotics are linked to an increased risk of falls, particularly in elderly patients, which is a significant concern given the widespread use of these medications in this population (37). Pharmacological treatments for insomnia have been evaluated primarily through short-term, placebo-controlled trials, and regulatory agencies have recently begun to require long-term data for medication approval (38, 39). As a result, pharmacological treatment is recommended mainly for the acute management of insomnia disorder and should not be the sole treatment for chronic insomnia (19–23). Furthermore, the comparative effectiveness of these medications remains unclear due to limited long-term evidence (40). An overview of the treatment guidelines for insomnia is presented in **Table 1**.

**Table 1:** Overview of treatment guidelines for insomnia (South Korea, United States, Europe).

	South Korea[22]		United States[23]	Europe[21]	
**Treatment Guideline**	Korean version of insomnia clinical practice guidelines: Diagnosis and treatment of insomnia (2019)		Management of Chronic Insomnia Disorder in Adults: A Clinical Practice Guideline From the American College of Physicians (2016)	The European Insomnia Guideline: An update on the diagnosis and treatment of insomnia (2023)	
**First-line treatment**	CBT-I		CBT-I	CBT-I (Face-to-face or digital)	
**Second-line treatment**	Isolated sleep onset insomnia	Sleep maintenance or mixed insomnia	- BZRA or Ramelteon - Sedating antidepressant	Short-term (≤4 weeks)	Long-term
	- Nonbenzodiazepine BZRA (zolpidem, zaleplon, eszopiclone*) - Benzodiazepine - Ramelteon*	- Z drug (zolpidem, zaleplon) - Doxepin, trazodone - Suvorexant* - Melatonin PR (≥55 yrs.)		- BZRA - Low dose sedating antidepressants - Daridorexant	- Daridorexant - Melatonin PR (≥55 yrs.)

CBT-I, cognitive behavioral therapy for insomnia; BZRA, benzodiazepine receptor agonist; PR, Prolonged Release.

*Ramelteon, suvorexant: Not on the market in South Korea; Eszopiclone: Introduced in November 2019 in South Korea.

Despite the established guidelines, there is a significant gap in understanding how these recommendations are implemented in real-world settings in South Korea. This study aims to provide a comprehensive description of current insomnia treatment practices in South Korea, focusing on both pharmacological and non-medication treatments. This includes examining treatment patterns and utilization rates associated with different therapeutic approaches. By offering a detailed overview of existing practices, this study seeks to identify gaps in implementing clinical guidelines and highlight areas for potential improvement.

## 2 Methods

The study was approved by the Institutional Review Board of Yonsei University (202306-HR-3406-01) and was conducted in accordance with the Declaration of Helsinki. As the data used were deidentified and retrospective, informed consent was waived.

### 2.1 Study design and data source

This population-based cohort study utilized data from the Korea National Health Insurance Service-National Sample Cohort (NHIS-NSC) database from 2002 to 2019. Since the introduction of universal health coverage in 1989, approximately 97% of the population has been enrolled in the NHIS. Utilizing systemic stratified sampling based on sex, age, residential location, insurance premium, and health insurance type, the NHIS-NSC included a representative sample of 1,000,000 individuals, making up to 2.2% of the national population [39]. The NHIS-NSC database comprises sociodemographic characteristics and diagnosis codes based on the International Classification of Diseases, 10th revision (ICD-10) (41), and healthcare utilization information derived from the National Health Insurance (NHI) claims for inpatient and outpatient visits. In detail, the NHI claims data contain principal and additional diagnoses, hospitalization and outpatient treatment details, prescribed medications, and health examination data. The classification codes for psychotherapy within the dataset are based on the setting in which the therapy was provided, such as individual therapy, group therapy, and family therapy. However, the database does not include specific codes for identifying the type of psychotherapy conducted, such as CBT-I or other specific therapeutic techniques.

### 2.2 Study population and cohort selection

**Figure 1:**
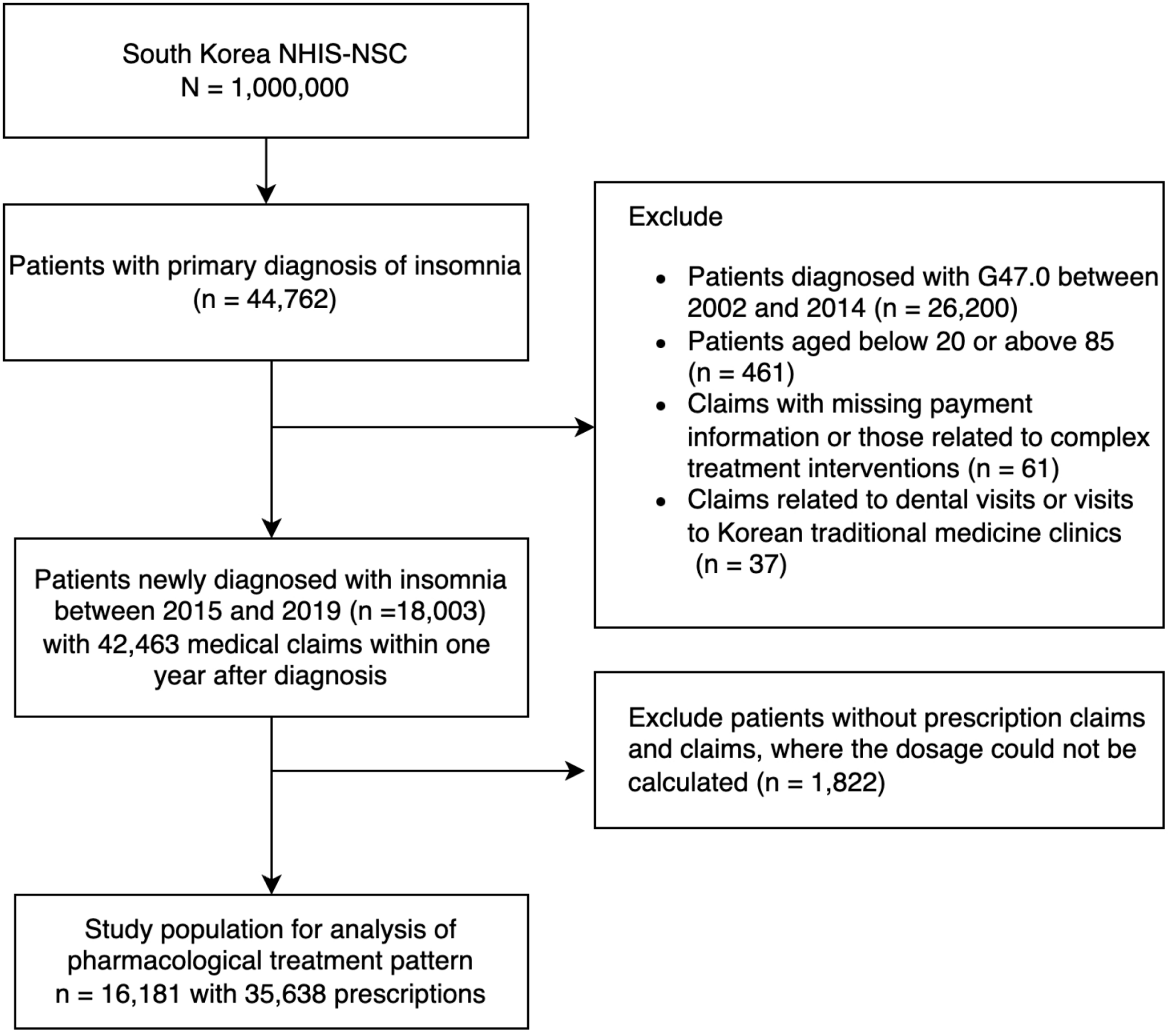
Flowchart showing the selection of study population.

We identified 44,762 patients with a primary diagnosis of insomnia (ICD-10 code G47.0) during outpatient visits, excluding those with primary diagnoses under ICD-10 ‘F codes’ to avoid confounding variables from psychiatric comorbidities. To focus on new cases of insomnia, we excluded patients diagnosed with G47.0 between 2002 and 2014 (n = 26,200). Additionally, we excluded patients aged younger than 20 years or older than 85 years (n = 461), those with missing payment information or those related to complex treatment interventions (n = 61), and those with claims related to dental visits or visits to Korean traditional medicine clinics (n = 37). This resulted in a cohort of 18,003 patients newly diagnosed with insomnia between 2015 and 2019, accounting for 42,463 medical claims within one year after diagnosis. To analyze pharmacological treatment patterns, we excluded 1,822 patients without prescription claims or for whom the dosage could not be calculated. The final study population comprised 16,181 patients with 35,638 prescriptions for insomnia medications. The selection process and exclusions are detailed in **Figure 1**.

### 2.3 Analysis

Patients were followed for one year after the diagnosis of insomnia. General characteristics are presented as frequencies (n) and percentages (%). Clinical outcomes within this period included the number of patients and claims, frequency of outpatient visits, and number of patients receiving psychotherapy. Under Korean medical law, medical institutions were classified based on bed count: clinics had fewer than 100 beds, and general hospitals had more than 100 beds (42).

For the pharmacological treatment analysis, we included medications prescribed or judged to be administered to improve insomnia symptoms. According to the clinical practice guidelines for insomnia published by the Korean Neuropsychiatric Association, 19 drugs listed as sleep medications registered in South Korea were included in this analysis (22). These medications were classified based on their mechanism of action into the following categories: short-acting benzodiazepines, intermediate-acting benzodiazepines, long-acting benzodiazepines, zolpidem (benzodiazepine-related drug, or “z-drugs”), antidepressants, melatonin and others (**Table 2**). The types of prescribed medications, duration of prescriptions, dosage and regimen of sleep medications, and patterns of dosage increases or decreases were analyzed. The defined daily dose (DDD) was calculated for each medication, following the World Health Organization (WHO) guidelines, representing the average maintenance dose per day for a drug used for its main indication in adults weighing 70 kg (43). Changes in DDD patterns were analyzed to identify shifts in prescribing practices, assess the impact of clinical guidelines, and evaluate trends over time.

**Table 2:** Medications for insomnia and their defined daily doses.

Mechanism of Action	Name	Anatomical Therapeutic Chemical	Defined Daily Dose (mg)
Benzodiazepine related drug	zolpidem	N05CF02	10
Short-acting Benzodiazepine	triazolam	N05CD05	0.25
Short-acting Benzodiazepine	etizolam	N05BA19	3
Intermediate Benzodiazepine	alprazolam	N05BA12	1
Intermediate Benzodiazepine	bromazepam	N05BA08	10
Intermediate Benzodiazepine	clonazepam	N03AE01	8
Intermediate Benzodiazepine	estazolam	N05CD04	3
Intermediate Benzodiazepine	lorazepam	N05BA06	2.5
Long-acting Benzodiazepine	diazepam	N05BA01	10
Long-acting Benzodiazepine	flunitrazepam	N05CD03	1
Antidepressant	trazodone	N06AX05	300
Antidepressant	amitriptyline	N06AA09	75
Antidepressant	mirtazapine	N06AX11	30
Antipsychotic	olanzapine	N05AH03	10
Antipsychotic	quetiapine	N05AH04	400
Others	doxepin	N06AA12	0.1
Others	agomelatine	N06AX22	25
Others	hydroxyzine	N05BB01	75
Others	melatonin	N05CH01	2

## 3 Results

### 3.1 Baseline characteristics

The study included 18,003 patients newly diagnosed with insomnia, generating 42,463 medical claims within one year. Of these, 16,181 patients received pharmacological treatment, resulting in 35,638 prescriptions. The majority were female (60.7% of the overall cohort and 60.6% of the pharmacological treatment cohort) and aged 40-65 years (47.3% and 47.1%, respectively), followed by those over 65 years (36.2% and 36.4%). Nearly half of the patients resided in metropolitan areas (47.6% and 46.9%). Low-income patients comprised 36.6% of the study population and 37.0% of the pharmacological treatment cohort. Most had a Charlson Comorbidity Index (CCI) of 0-2 (58.8% and 58.8%), indicating a low level of comorbid conditions (**Table 3**).

**Table 3:** Baseline characteristics of the overall cohort and pharmacological treatment cohort.

	Overall cohort				Pharmacological treatment cohort			
	n = 18003	%	42463 Claims*	%	n = 16181	%	35638 Claims*	%
Sex								
-Male	7074	39.29	17333	40.82	6375	39.40	14792	41.51
-Female	10929	60.71	25130	59.18	9806	60.60	846	2.37
Age (years)								
-20-40	2978	16.54	7443	17.53	2666	16.48	6516	18.28
-40-65	8506	47.25	18759	44.18	7620	47.09	15770	44.25
->65	6519	36.21	16261	38.29	5895	36.43	13352	37.47
Region								
-Metropolitan	8563	47.56	20697	48.74	7586	46.88	16859	47.31
-Urban	3535	19.64	8251	19.43	3230	19.96	7193	20.18
-Rural	5905	32.80	13515	31.83	5365	33.16	11586	32.51
Income								
-Low	6592	36.62	15875	37.39	5987	37.00	13645	38.29
-Middle	6186	34.36	14416	33.95	5560	34.36	12240	34.35
-High	5225	29.02	12172	28.66	4634	28.64	9753	27.37
CCI								
-0-2	10590	58.82	24877	58.59	9520	58.83	21094	59.19
-3 - 5	5796	32.19	13604	32.04	5206	32.17	11300	31.71
->5	1617	8.98	3982	9.38	1455	8.99	3244	9.10

*Number of claims made within one year after diagnosis.

### 3.2 Insomnia patient distribution and psychotherapy utilization trends

**Figure 2:**
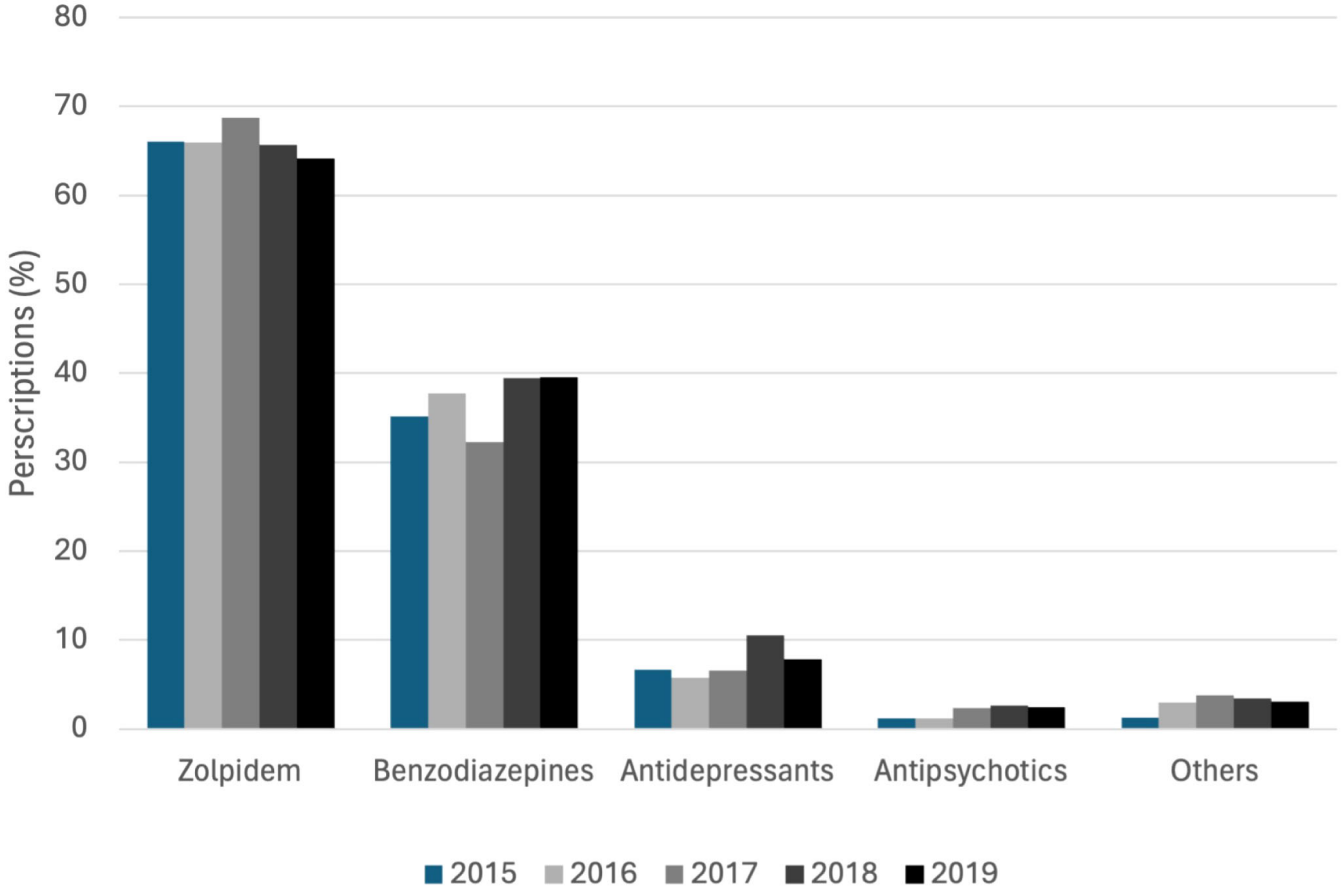
Number of insomnia patients and claims per person by year. Distribution of insomnia patients treated in clinics and general hospitals from 2015 to 2019, along with the average number of claims per person each year.

Most insomnia patients were treated in clinics, with approximately 3,500 patients treated annually from 2015 to 2019. In 2015, 3,549 patients were treated, with 90.3% in clinics and 9.7% in general hospitals. By 2019, 3,762 patients were treated, 87.1% in clinics and 12.9% in general hospitals. The number of claims per person varied slightly over the years, peaking at 2.6 in 2018. **Figure 2** illustrates the distribution of the number of patients between clinics and general hospitals, as well as the number of claims per person each year.

**Figure 3:**
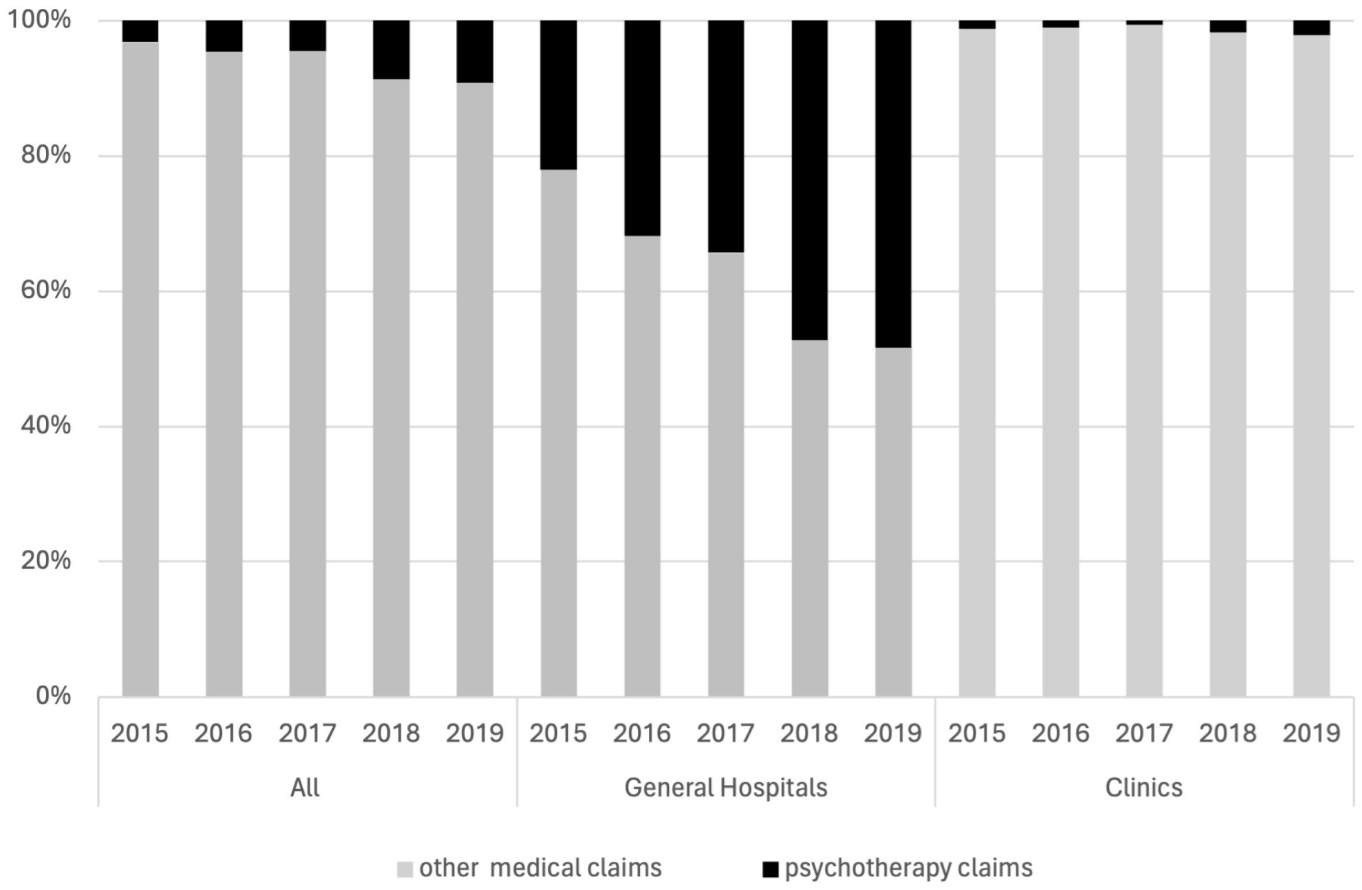
Psychotherapy claims for insomnia by year and setting. Percentage of psychotherapy claims relative to all medical claims for insomnia patients from 2015 to 2019, differentiated by general hospitals and clinics.

The number of visits for psychotherapy in the first year after insomnia diagnosis was analyzed from 2015 to 2019. Psychotherapy claims included individual psychotherapy, group psychotherapy, and family therapy. The overall proportion of psychotherapy claims relative to all medical claims increased steadily during this period. In 2015, 3.2% of all medical claims were for psychotherapy, which rose to 9.1% by 2019. In general hospitals, psychotherapy claims constituted 22.1% of the total claims in 2015, increasing to 48.4% in 2019. In clinics, however, the proportion of psychotherapy claims was low, starting at 1.3% in 2015 and rising to 2.1% in 2019 (**Figure 3**).

### 3.3 Pharmacological treatment of insomnia

The number of medical claims, prescriptions for pharmacological treatment, and prescription rates from 2015 to 2019 are summarized in **Table 4**. The prescription rate remained consistently high, from 86.8% to 89.4% over the years.

**Table 4:** Medical Claims and Prescription Rates for Pharmacological Treatment.

Year	Medical claims (A)	Prescription for pharmacological treatment	
		Number of claims (B)	Prescription rate (%)
2015	8,657	7,517	86.83
2016	8,592	7,682	89.41
2017	8,357	7,461	89.28
2018	9,613	8,501	88.43
2019	7,244	6,404	88.40

**Figure 4:**
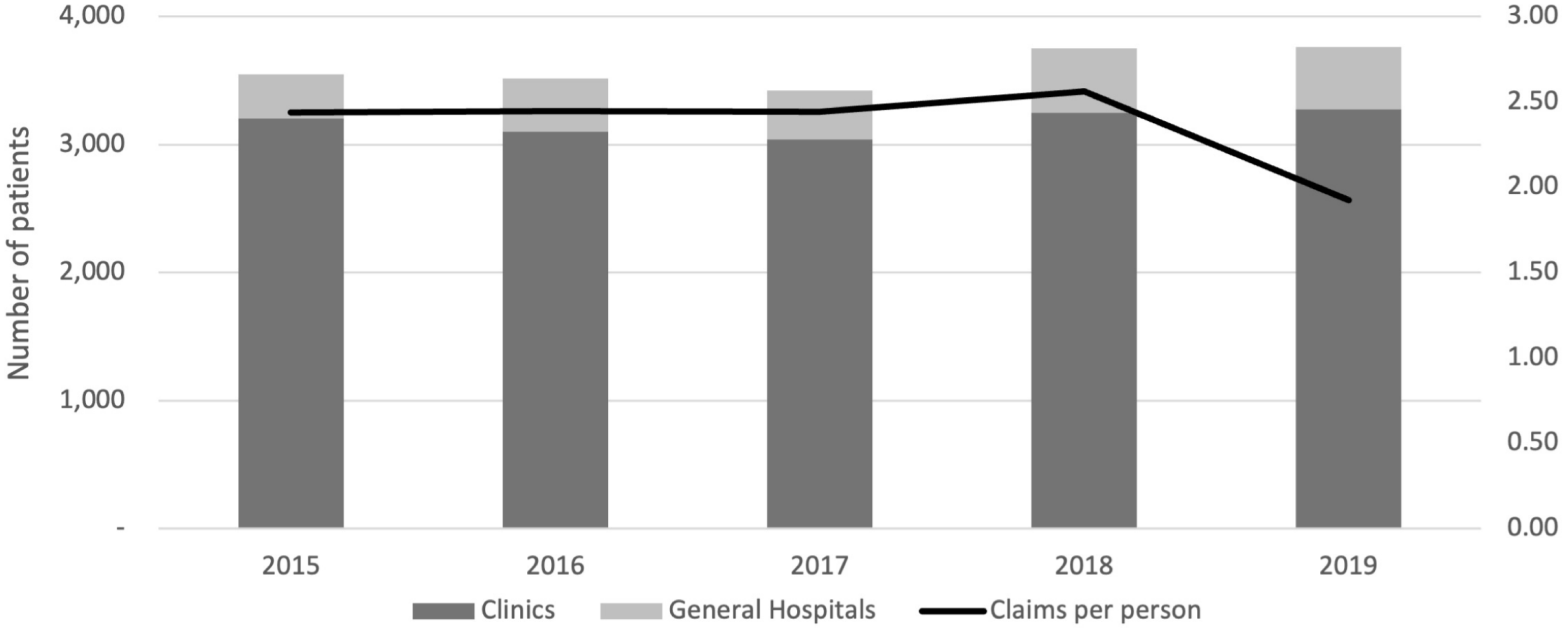
Distribution of medication classes prescribed to insomnia patients from 2015 to 2019. The graph shows the percentage of patients with zolpidem, benzodiazepines, antidepressants, antipsychotics, and other medications each year.

The analysis of pharmacological treatments for insomnia from 2015 to 2019 revealed the distribution of various medication classes prescribed. Zolpidem was the most prescribed class of medication, accounting for more than 60% of prescriptions each year. Benzodiazepines were the second most frequently prescribed, with their usage ranging between approximately 30% and 40%. The use of antidepressants slightly increased over time, while the use of antipsychotics and other medications remained at relatively low and stable levels (**Figure 4**).

### 3.4 Outpatient visit-based prescription duration and DDD

**Figure 5:**
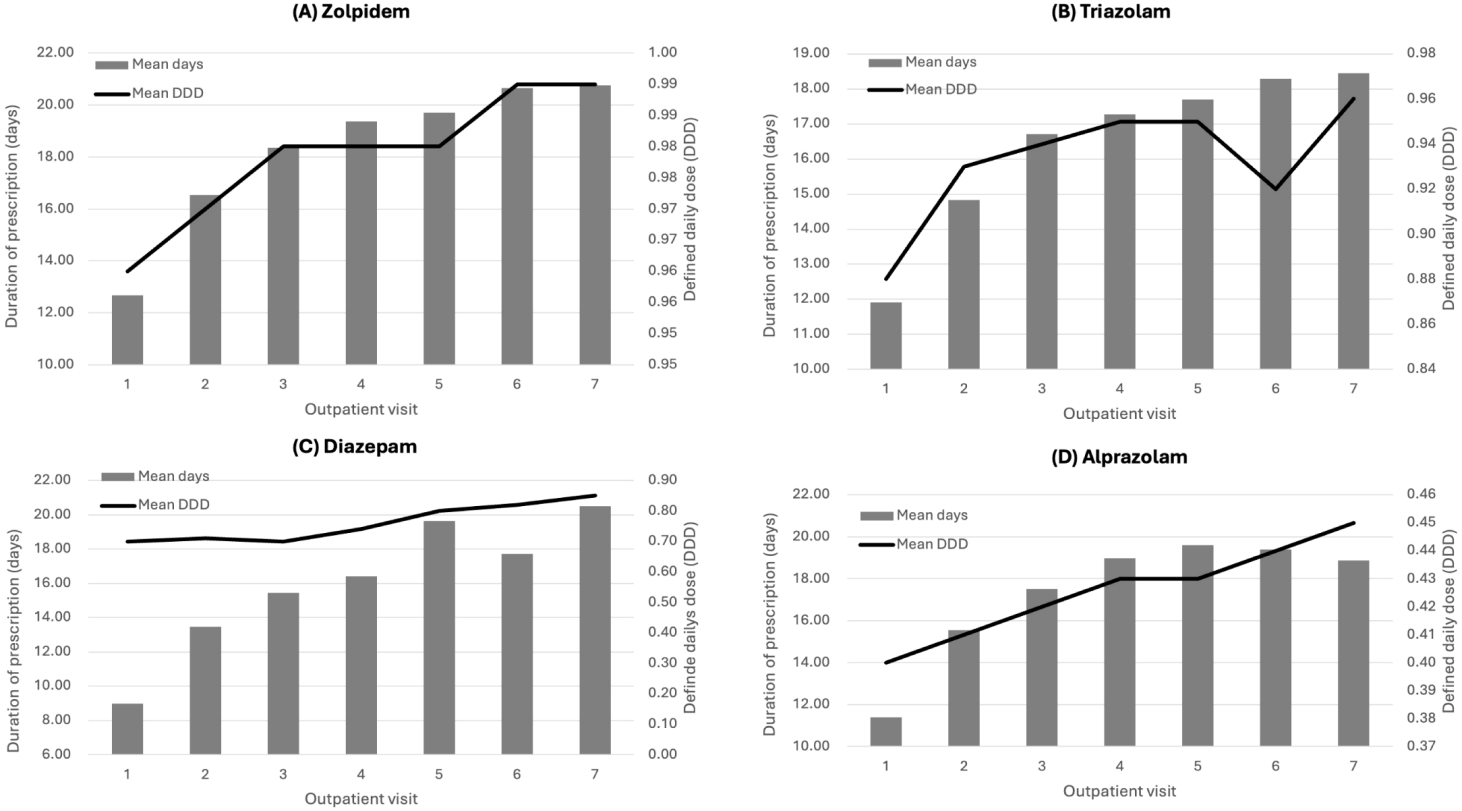
Duration of prescriptions and mean DDD by outpatient visit for **(A)** Zolpidem, **(B)** Triazolam, **(C)** Diazepam, and **(D)** Alprazolam.

The analysis of outpatient visit-based prescription duration DDD was conducted for zolpidem, triazolam, diazepam, and alprazolam. **Figure 5** illustrates the mean duration of prescription and DDD across different outpatient visits for these medications. The figure shows the average duration of prescriptions (in days) and the mean DDD for zolpidem (A), triazolam (B), diazepam (C), and alprazolam (D) across different outpatient visits. Zolpidem had an initial prescription duration of 12.8 days, which increased to 20.8 days by the seventh visit, with a stable mean DDD close to 1.0, indicating consistent dosing practices despite longer prescription durations. Triazolam exhibited a similar trend, with the duration increasing from 11.9 days at the first visit to 18.4 days by the seventh visit. The mean DDD remained relatively stable, around 0.9, with slight fluctuations observed across visits. Diazepam showed an increase in prescription duration from 9.0 days to 20.5 days over seven visits. The mean DDD for diazepam was the lowest among the four medications, starting at approximately 0.7 and gradually increasing to 0.9 by the seventh visit, reflecting consistent but gradually increasing dosing. Alprazolam shows increasing durations from around 11.4 to 19.0 days, with a consistent mean DDD of around 0.4. Overall, the analysis indicates that while the duration of prescriptions for these medications increases with successive outpatient visits, the mean DDD for each drug remains relatively stable, with minor variations. This stability in DDD suggests consistent dosing practices across different patient visits, even as the duration of treatment extends over time.

## 4 Discussion

This study analyzed the treatment patterns and utilizations of patients newly diagnosed with insomnia using data from the Korea National Health Insurance Service-National Sample Cohort (NHIS-NSC) from 2015 to 2019.

Our analysis indicated that the majority of insomnia patients in South Korea were treated in clinics, with pharmacological treatment being the predominant management strategy. Zolpidem and benzodiazepines were the most frequently prescribed medications, accounting for a substantial portion of all prescriptions. The analysis of prescription patterns revealed consistent dosages with increasing treatment lengths across outpatient visits. Specifically, the DDD for commonly used insomnia medications such as zolpidem, triazolam, diazepam, and alprazolam remained stable, even as the duration of prescriptions increased with each subsequent outpatient visit. This pattern suggests that while clinicians are extending the duration of treatment, they are maintaining consistent dosing practices. However, it Is Important to note that current clinical guidelines recommend that both Z-drugs and benzodiazepines be used only for short-term treatment of insomnia, typically limited to two to four weeks (18, 20, 44). Prolonged use of these medications carries significant risks, including the development of dependence, cognitive impairment, and psychomotor impairment, which can lead to falls and fractures, particularly in elderly patients (36, 37). These findings are consistent with international trends despite the recommendations of clinical guidelines (18, 45). The observed extension of prescription durations in our study indicates a potential deviation from recommended practices. This highlights the need for clinicians to carefully assess the benefits and risks of continued use of Z-drugs and benzodiazepines, ensure that treatment duration is minimized, and follow appropriate tapering protocols to mitigate the risks associated with long-term use (44, 46).

Among the “Z-drugs,” eszopiclone has been shown to have the best efficacy and acceptability profiles for both short-term and long-term use (18). However, it was not included in our analysis due to its late market entry (at the end of 2019) in South Korea. Novel pharmacological treatments that are not yet available in South Korea include medications targeting new mechanisms, such as ramelteon (facilitating melatonin activity), and orexin receptor antagonists, such as daridorexant, lemborexant, seltorexant, and suvorexant (47). Lemborexant has demonstrated strong efficacy for both short-term and long-term treatment of insomnia (18). These advancements represent potential future options for insomnia management in South Korea.

Our findings indicate that despite international guidelines recommending CBT-I as the first-line treatment for insomnia, its utilization, as inferred from general psychotherapy claims, remains low, particularly in clinics. This observation is consistent with previous research, which has shown that the adoption of CBT-I in routine practice is limited despite its proven efficacy (24, 26, 32). In July 2018, the Ministry of Health and Welfare in South Korea included psychotherapy under the coverage of NHIS as part of efforts to diversify the payment system for psychiatric treatments. This policy change may account for the increased psychotherapy claims observed in 2018 and 2019, mainly in general hospitals. However, despite this expansion in coverage, the overall utilization of psychotherapy, and by extension, CBT-I, continues to be limited, suggesting that further efforts are necessary to promote its adoption. Barriers to the broader use of CBT-I include a shortage of trained professionals, time constraints, and patient preferences for quicker, medication-based solutions (35, 45, 48). New modalities, such as digital CBT-I, show promise in enhancing accessibility and utilization by offering a convenient alternative to traditional therapy (31).

The comprehensive data from the NHIS-NSC allowed for an analysis of prescription patterns and healthcare utilization, providing an analysis of insomnia management in South Korea. However, this study has several limitations. A key limitation of this study is the NHIS-NSC database’s lack of a specific code for CBT-I, which prevents precise identification of this treatment within psychotherapy claims. This means that while we observe a general increase in psychotherapy utilization, we cannot ascertain the extent to which evidence-based therapies like CBT-I are being implemented. Additionally, as a descriptive analysis, no associations were made between specific groups of patients and their utilization of treatment methods. The reliance on insurance claims data may lead to underreporting specific treatments, particularly non-pharmacological interventions such as psychotherapy, which can be conducted outside the medical setting. These sessions are not included in insurance claims data. Furthermore, the exclusion criteria, particularly for patients with psychiatric comorbidities, limit the generalizability of the findings to all insomnia patients. Also, the presence of comorbid sleep disorders, such as obstructive sleep apnea (OSA), was not analyzed, although comorbid insomnia and sleep apnea (COMISA) is prevalent and complicates insomnia management, affecting up to 35% of insomnia patients (49, 50).

Future research should aim to explore the risks based on demographics and investigate the relationships with therapy utilization. Additionally, comparisons between the psychotherapy and pharmacological treatment groups could help identify potential referral biases and predictors for psychotherapy treatment. Studies focusing on the barriers to psychotherapy utilization could provide insights into improving adherence to clinical guidelines. Moreover, research incorporating a broader range of comorbid conditions would offer a more comprehensive view of insomnia management in diverse patient populations.

In conclusion, our study highlights the predominant use of pharmacological treatments for insomnia and the underutilization of psychotherapy despite its clinical recommendations. These findings suggest a need for enhanced efforts to integrate psychotherapy into routine clinical practice and further research to optimize insomnia management strategies.

## Data Availability

Publicly available datasets were analyzed in this study. This data can be found here: https://nhiss.nhis.or.kr/.
